# Subclinical iron deficiency is a strong predictor of bacterial vaginosis in early pregnancy

**DOI:** 10.1186/1471-2334-5-55

**Published:** 2005-07-06

**Authors:** Hans Verstraelen, Joris Delanghe, Kristien Roelens, Stijn Blot, Geert Claeys, Marleen Temmerman

**Affiliations:** 1 Department of Obstetrics & Gynaecology, Faculty of Medicine and Health Sciences, Ghent University, Ghent University Hospital, De Pintelaan 185, B-9000 Ghent, Belgium; 2 Department of Clinical Chemistry, Microbiology and Immunology, Faculty of Medicine and Health Sciences, Ghent University, Ghent University Hospital, De Pintelaan 185, B-9000 Ghent, Belgium; 3 Department of Intensive Care, Faculty of Medicine and Health Sciences, Ghent University, Ghent University Hospital, De Pintelaan 185, B-9000 Ghent, Belgium

## Abstract

**Background:**

Bacterial vaginosis (BV) is the single most common vaginal infection in women of childbearing age and associated with a sizeable infectious disease burden among both non-pregnant and pregnant women, including a significantly elevated risk of adverse pregnancy outcome. Overall, little progress has been made in identifying causal factors involved in BV acquisition and persistence. We sought to evaluate maternal iron status in early pregnancy as a putative risk factor for BV, considering that micronutrients, and iron deficiency in particular, affect the host response against bacterial colonization, even in the setting of mild micronutrient deficiencies.

**Methods:**

In a nested case-control study, we compared maternal iron status at entry to prenatal care (mean gestational age 9.2 ± 2.6 weeks) between eighty women with healthy vaginal microflora and eighteen women with vaginosis-like microflora. Vaginal microflora status was assessed by assigning a modified Nugent score to a Gram-stained vaginal smear. Maternal iron status was assayed by an array of conventional erythrocyte and serum indicators for iron status assessment, but also by more sensitive and more specific indicators of iron deficiency, including soluble transferrin receptors (sTfR) as an accurate measure of cellular and tissue iron deficiency and the iron deficiency log_10_[sTfR/ferritin] index as the presently most accurate measure of body storage iron available.

**Results:**

We found no statistically significant correlation between vaginal microflora status and routinely assessed iron parameters. In contrast, a highly significant difference between the healthy and vaginosis-like microflora groups of women was shown in mean values of sTfR concentrations (1.15 ± 0.30 mg/L versus 1.37 ± 0.38 mg/L, p = 0.008) and in mean iron deficiency log_10_[sTfR/ferritin] index values (1.57 ± 0.30 versus 1.08 ± 0.56, p = 0.003), indicating a strong association between iron deficiency and vaginosis-like microflora. An sTfR concentration >1.45 mg/L was associated with a 3-fold increased risk (95%CI: 1.4–6.7) of vaginosis-like microflora and after controlling for maternal age, gestational length, body mass, parity, and smoking habits with an adjusted odds ratio of 4.5 (95%CI: 1.4–14.2).

**Conclusion:**

We conclude that subclinical iron deficiency, presumably resulting from inadequate preconceptional iron supplies, is strongly and independently associated with vaginosis-like microflora during early pregnancy.

## Background

Bacterial vaginosis (BV) is the single most common vaginal infection in women of childbearing age. Basically, BV involves a shift from the predominant hydrogen peroxide-producing *Lactobacillus *species, such as *L. crispatus *and *L. jensenii *[1-3] to a polymicrobial flora that includes gram-variable and gram-negative anaerobes such as *Gardnerella vaginalis*, *Prevotella *spp., and *Mobiluncus *spp. [4,5], and more recently associated gram-positive anaerobes such as *Peptostreptococcus *spp. [6] and *Atopobium vaginae *[3,7-9].

Besides a nuisance problem causing vaginal discomfort [10], BV is also associated with a sizeable disease burden [11]. In pregnancy, the presence of vaginosis-like microflora especially during early gestation has been consistently and strongly associated with spontaneous preterm labour and preterm prelabour rupture of membranes, and hence, BV is a major determinant to the prematurity-related disease burden [12]. In addition, though not considered a conventionally defined sexually transmitted infection (STI), BV strongly predisposes to the acquisition of pandemic STIs, such as *Neisseria gonorrhoeae *[13], *Chlamydia trachomatis *[13], and most notably HIV-1 among both pregnant [14,15] and non-pregnant women [16-19].

A few factors are known to increase the risk of BV, including demographic factors such as black ethnicity, and behavioural factors including smoking, vaginal douching, IUD contraceptive use, and sexual behaviour-related factors [20]. Overall, little progress has been made however in identifying causal factors involved in BV acquisition and recurrence [21,22]. In particular, there is a striking dearth of data on intrinsic or biological risk factors for BV. Nonetheless, since BV is not deemed a traditionally defined STI, it is all the more likely that intrinsic factors elicit a pivotal role in the acquisition of disturbed vaginal microflora and, in some women, are underlying the apparent instability of the vaginal ecosystem. In particular, it is increasingly assumed that subtle differences in the type and magnitude of the host-pathogen response at the level of the vaginal mucosa may explain the differential susceptibility to perturbation of the vaginal niche [23]. Some very recent findings in the field of the innate vaginal mucosal immune response, including the demonstration of genetic differences, such as Toll 4-like receptor gene mutations [24], and of phenotypic differences such as impaired expression of anti-inflammatory cytokines [25], being strongly correlated with the susceptibility to bacterial vaginosis are unique examples to the above paradigm.

From this perspective, we hypothesized that micronutrient status during early pregnancy may represent yet another putative biological risk factor for BV, considering that micronutrients, and iron deficiency in particular, affect the host response against bacterial colonization [26], even in the setting of mild micronutrient deficiencies [27]. Moreover, maternal iron deficiency has been consistently associated with adverse pregnancy outcome [28,29] and it has therefore been reiterated that micronutrient status during early pregnancy warrants further scrutiny even among well-nourished women from high-income countries [28].

## Methods

### Study population and design

As part of a prospective cohort study basically involving the study of vaginal microflora during early pregnancy in relation to pregnancy outcome, we conducted a nested case-control study comprising 115 unselected pregnant women, which were consecutively enrolled on the occasion of their first antenatal visit, between March 3 and November 6, 2003 at the outpatient obstetric clinic of the Ghent University Hospital. The Ghent University Hospital Ethical Board approved the study protocol and all study subjects agreed to participate through written informed consent.

### Sample collection

Vaginal samples were collected for the purpose of vaginal microflora status assessment by inserting a sterile cotton-tipped wooden swab into the vagina. The swab was rolled round through 360 degrees against the vaginal wall at the midportion of the vault and carefully withdrawn to prevent contamination. Swabs were then smeared on a plain glass slide and air-dried at room temperature. The slides were Gram stained and assigned a modified *Nugent *score [4] according to *Ison *and *Hay *[30]. Accordingly, Gram-stained vaginal smears were initially categorized as normal (grade I), intermediate (grade II), and bacterial vaginosis (grade III). To the purpose of the present study, we subsequently pooled the latter two categories into a single category unless otherwise specified, and therefore further denote two vaginal microflora status categories: normal or healthy microflora (corresponding to grade I microflora or a Nugent score 0–3) and disturbed or bacterial vaginosis-like microflora (corresponding to grade II and III or a Nugent score 4–10).

Blood samples were drawn within one hour following vaginal swabbing and processed within four hours. A first venous blood sample was allowed to clot, and centrifuged (1000 *g *for 10 minutes at room temperature). The supernatant serum was collected for analysis. Serum ferritin, soluble transferrin receptors (sTfR), and C-reactive protein (CRP) were assayed by fixed-time immunonephelometry using commercial rabbit anti-human antisera on a BN II nephelometer (*Dade Behring*), calibrated against the CRM 470 certified reference material. Serum transferrin concentration was assessed by immunoturbidimetry using commercial reagents on a Modular P analyzer (*Roche Diagnostics*). Serum iron concentration was measured by spectrophotometry (ferrozine method). Serum transferrin saturation (TS) was calculated as TS (%) = [serum iron (μmol/L)/serum transferrin (g/L)] × 3.98.

Additional indices of iron deficiency were calculated following log_10 _transformation of serum ferritin and sTfR concentrations, including the log_10 _[sTfR]/log_10_[ferritin] and log_10 _[sTfR/ferritin] indices [31]. These combined measures have recently evolved as highly specific and sensitive measures of iron deficiency [31]. In particular, the logarithm of the ratio of the soluble transferrin receptor to ferritin concentration (log_10 _[sTfR/ferritin]) is currently the most precise measure of body storage iron available [32,33].

A second venous blood sample was simultaneously collected in EDTA tubes to assess plasma haemoglobin (Hgb), red blood cell count (RBC) and haematocrit (Hct), mean corpuscular volume (MCV) and mean corpuscular haemoglobin (MCH) (*Sysmex SE-9500; Toa Medical Electronics*).

All women had a vaginal ultrasound (US) scan and if embryonal biometry was inconsistent with the calculated gestational length, the latter was adjusted according to US biometry. Body mass index or BMI (kg/m^2^) was calculated as weight (kg)/[length (m)] ^2 ^after standardized assessment of maternal weight and length at entry to prenatal care. All other clinical data were collected in a routine manner.

### Exclusion criteria

Exclusion criteria were first attendance beyond fourteen completed weeks of gestation, multiple gestation, and pre-existing maternal systemic disease, e.g. diabetes mellitus. Women who were unacquainted with the Dutch language could not be included because we had no means to obtain informed consent from these in a proper way.

### Definitions

For initial assessment of subjects, iron deficiency and anaemia were defined according to the WHO criteria of serum ferritin ≤ 12 g/L and haemoglobin < 11 g/dL, respectively. Iron deficiency was further investigated by the use of more sensitive indicators, in particular maternal sTfR concentrations and the log_10 _[sTfR/ferritin] index; however, no reference values for pregnant women are available at present for these parameters.

### Statistical analyses

Following the one-sample Kolmogorov-Smirnov test procedure for each variable, we established that all continuous variables could be analysed under the parametric assumption (p-value to the Z-statistic >0.5), except maternal serum CRP. Means are presented as arithmetic means and standard deviation to the mean. Means between two groups were compared with the independent samples *t *test. Strength of bivariate correlations was expressed as Pearson correlation coefficients (R). Strength of association was calculated as prevalence or risk ratios (RR) in a univariate analysis and (adjusted) odds ratios (OR) in a multivariable analysis with 95% confidence intervals (CI) and p-values to the 95% CI. Multivariable analysis was performed using a stepwise binary logistic regression model and likelihood ratio tests were used to compare different models. For any reported measure, statistical significance was accepted, as the two-tailed probability level was <0.05.

All statistical analyses were performed using the statistical software package SPSS v12.0 (*Chicago*, *Illinois*).

## Results

### Study population

We excluded seventeen women (17/115 or 14.8%) who had given their consent for participation in the study, from the analysis, because they refused part of the sampling procedure, or because of incomplete or inadequate sampling, including delay between vaginal and blood sampling, or because of early (missed) abortion. As a group, the subjects excluded did not differ significantly from the remainder of women in terms of baseline characteristics (mean maternal age, mean body mass index, smoking habits, mean gestational age, and median parity and gravidity).

From each patient included for the final analysis (98/115 or 85.2%) a vaginal swab and venous blood samples were obtained at a single point in time at a mean gestational age of 9.2 ± 2.6 weeks. Eighty women had normal or grade I microflora on Gram stain and eighteen women presented with disturbed or vaginosis-like microflora, i.e. intermediate (grade II) microflora or overt bacterial vaginosis (grade III) microflora.

Basic clinical characteristics of study participants, who were all of white Caucasian origin, are displayed in table 1. Women with disturbed vaginal flora in the index pregnancy were significantly more likely to have delivered a child previously and tended to have a higher body mass index (Table 1).

**Table 1:** Basic clinical characteristics of the study population.

	Healthy vaginal microflora (n = 80)	Disturbed vaginal microflora (n = 18)	p-value
Maternal age (years) – mean ± SD	30.5 ± 4.7	30.2 ± 5.7	0.8
Gestational age (weeks) – mean ± SD	9.3 ± 2.6	8.7 ± 2.8	0.4
Body mass index (kg/m^2^) – mean ± SD	22.7 ± 4.0	24.9 ± 5.8	0.05
Body mass index (kg/m^2^) – % (n)			
<25	77.5% (62)	50.0% (9)	0.04
≥25	22.5% (18)	50.0% (9)	
Parity – % (n)			
0	43.8% (35)	16.7% (3)	0.04
≥1	56.3% (45)	83.3% (15)	
Gravidity – % (n)			
0	35.0% (28)	11.1% (2)	0.05
≥1	65.0% (52)	88.9% (16)	
Smoking – % (no)			
Yes	11.1% (2)	22.2% (4)	0.1
No	88.9% (16)	77.8% (14)	

Healthy vaginal flora is defined as grade I or lactobacilli-dominated microflora on Gram stain (corresponding to a Nugent score 0 – 3) and disturbed vaginal flora is defined as grade II and grade III flora, this is mixed or gram-negative rods-dominated microflora on Gram stain (corresponding to a Nugent score ≥4). Body mass index (BMI) refers to the BMI at entry to prenatal care and is calculated as weight (kg)/[length (m)]^2^.

### Traditional indicators of iron deficiency in relation to vaginal microflora status

In this study sample, 10.2% of subjects (10/98) had depleted iron stores during early pregnancy according to the conventional criterion of serum ferritin ≥ 12 g/L. 4.1% of women (4/98) presented with anaemia defined as Hgb < 11 g/dL and 10.2% with a Hgb concentration below 12 g/dL (10/98), though only one of these (1/98 or 1.0%) had true iron-deficient anaemia when accounting for both serum ferritin (≥ 12 g/L) and haemoglobin (< 11 g/dL). Any other traditional compound indices of iron-deficiency, e.g. by accounting for mean cell volume, mean corpuscular haemoglobin, and transferrin saturation did not identify any additional cases of clinical overt iron deficiency.

There was no statistically significant correlation between vaginal microflora status as assessed by Gram stain and any of the conventionally assessed iron and red blood cell indices, including red blood cell counts, serum haemoglobin, haematocrit, mean corpuscular volume, mean corpuscular haemoglobin, serum iron, serum ferritin, serum transferrin and transferritin saturation (Table 2). Similarly, we observed no significant association between vaginal microflora status and maternal CRP concentration during early pregnancy. In this cohort there were no cases of overt systemic inflammation according to maternal CRP, nor were ferritin and transferrin concentrations significantly correlated with CRP.

**Table 2:** Maternal serum iron and red blood cell indices according to vaginal microflora status. Healthy vaginal flora is defined as grade I or lactobacilli-dominated microflora on Gram stain (corresponding to a Nugent score 0 – 3) and disturbed vaginal flora is defined as grade II and grade III flora, this is mixed or gram-negative rods-dominated microflora on Gram stain (corresponding to a Nugent score ≥4). RBC denotes red blood cell counts, Hgb plasma haemoglobin concentration, Hct hematocrit, MCV mean corpuscular volume of red blood cells, MCH mean corpuscular haemoglobin mass of red blood cells, Fe serum iron concentration, and sTfR (serum) soluble transferrin receptor concentration, respectively.

	Healthy vaginal microflora (n = 80)	Disturbed vaginal microflora (n = 18)	p-value
RBC (×10^6^/μL)	4.3 ± 0.4	4.4 ± 0.4	0.5
HgB (g/dL)	13.1 ± 0.9	13.1 ± 1.1	0.8
Hct (%)	38.8 ± 2.6	39.4 ± 3.2	0.4
MCV (fL)	89.4 ± 4.6	89.1 ± 5.3	0.8
MCH (pg/cell)	30.2 ± 1.8	29.7 ± 1.5	0.3
Fe (μg/dL)	113.0 ± 39.1	105.2 ± 43.3	0.5
Ferritin (mg/L)	46.2 ± 35.9	52.5 ± 56.6	0.6
Transferrin (mg/dL)	2.8 ± 0.5	2.9 ± 0.5	0.7
Transferrin saturation (%)	29.5 ± 12.1	26.7 ± 12.3	0.4

sTfR (mg/L)	1.15 ± 0.30	1.37 ± 0.38	0.01
Log_10_[sTfR]/log_10_[ferritin]	0.04 ± 0.12	0.10 ± 0.15	0.04
Log_10_[sTfR/ferritin]	1.57 ± 0.30	1.08 ± 0.56	0.003

### Soluble transferrin receptors in relation to vaginal microflora status

**Figure 1:**
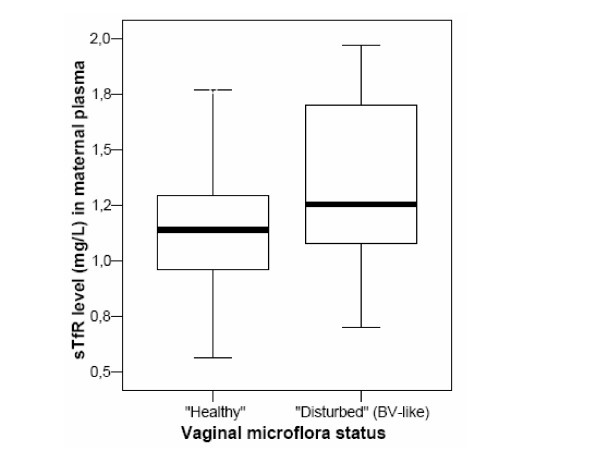
**Distribution of sTfR concentrations according to vaginal microflora status**. Box-and-whisker plots of the sTfR distributions according to vaginal microflora status during early pregnancy. The thick line represents the median sTfR value, the horizontal box lines the 25^th ^percentile and 75^th ^percentile, and the outer short horizontal lines the boundaries of the sTfR range. Healthy vaginal flora is defined as grade I or lactobacilli-dominated microflora on Gram stain (corresponding to a Nugent score 0 – 3) and disturbed vaginal flora is defined as grade II and grade III flora, this is mixed or gram-negative rods-dominated microflora on Gram stain (corresponding to a Nugent score ≥4).

In contrast to the above, we observed a trend by which maternal serum transferrin receptor (sTfR) concentrations during early pregnancy were negatively correlated with lactobacillary grading and hence positively correlated with the degree of vaginal microflora alteration (R = 0.26, p = 0.01). When vaginal microflora status was handled as a dichotomous variable ('healthy' versus 'disturbed'), women with healthy vaginal microflora (n = 80) during early pregnancy had a mean sTfR concentration of 1.15 ± 0.30 mg/L as compared to a mean sTfR of 1.37 ± 0.38 mg/L among women (n = 18) with disturbed microflora (p = 0.008) (Figure 1).

Given the significant overlap in sTfR distributions for the healthy and disturbed vaginal microflora groups of women, classification plots were constructed and the sTfR value with the highest discriminative value between both groups was chosen as the sTfR cut-off level. Serum transferrin receptor concentrations > 1.45 mg/L were associated with a prevalence or risk ratio of 3.0 (95% CI: 1.4 – 6.7, p = 0.014) for disturbed vaginal flora. The accuracy of the sTfR assay at this threshold was 79% (95% CI: 0.69–0.86). Sensitivity and the positive predictive value (PPV) were however low and estimated at 39% (95% CI: 0.18–0.64) and 41% (95% CI: 0.19–0.67), respectively. In contrast, specificity and consequently the negative predictive value (NPV) of the assay were as high as 88% (95% CI: 0.78–0.94) and 86% (95% CI: 0.77–0.93), respectively.

Of the parameters entered into in the multivariable analysis only smoking, body mass index and gravidity/parity were expected to act as true confounders of the association under study considering these variables impinge both on serum transferrin receptor concentrations and on vaginal microflora status. We also controlled for maternal age, gestational age at sampling, and CRP as covariates in the model. Yet, the only significant variable retained from the multivariable analysis was the maternal sTfR concentration, suggesting that raised sTfR concentrations during early pregnancy are independently associated with vaginal microflora alteration. The adjusted odds ratio of an sTfR-concentration > 1.45 mg/L for vaginosis-like vaginal microflora was 4.5 (95%CI: 1.4–14.2, p = 0.011).

### The log_10 _[sTfR/ferritin] iron deficiency index in relation to vaginal microflora status

Since sTfR concentrations may reflect both rates of erythropoiesis as well as cellular iron needs, several combined measures following log transformation of sTfR and/or ferritin have been used as highly specific and sensitive measures of iron deficiency in particular, including sTfR/log_10_[ferritin], log_10_[sTfR]/log_10_[ferritin], and log_10_[sTfR/ferritin] [31].

The logarithm of the ratio of the soluble transferrin receptor to ferritin concentration (log_10 _[sTfR/ferritin]) is currently the most precise measure of body storage iron available [32,33]. We found a highly significant difference (difference in means = 0.49, 95% CI 0.30–0.68, p < 0.0001) between mean values of the log_10 _[sTfR/ferritin] index (1.57 ± 0.30 versus 1.08 ± 0.56) between both groups (Table 2) further indicating that the observed association between maternal sTfR concentrations and vaginal microflora status indeed relates to depleted available body iron stores and cellular iron avidity.

Though our sample size was rather small, post hoc analysis revealed that the above difference in the mean iron deficiency index values (log_10_[sTfR/ferritin]) between both groups was demonstrated at a two-sided significance level of α = 0.05 with a statistical power of more than 90% (1-β = 92.7) under the parametric assumption and by accounting for unequal variances.

### Supplemental iron intake as a potential determinant of iron indices

Of note is that 65.3% of women in this cohort (64/98) were already taking oligo-elements or vitamin supplements at the time of sampling, most often as combined preparations (35/98 or 35.7%) or as folate supplements (27/98 or 27.6%). We found no significant association between iron supplementation (37/98 or 37.8%) and maternal sTfR concentrations (p = 0.95) or with the combined sTfR-ferritin indexes following log transformation (p = 0.20 to 0.97), while there was a marginally significant association between serum ferritin concentrations and supplemental iron intake (p = 0.053).

## Discussion

### The association of subclinical iron deficiency with vaginosis-like microflora

We found that maternal serum concentrations of soluble transferrin receptors during early pregnancy were positively correlated with decreased lactobacillary grading and hence with degree of vaginal microflora alteration. An sTfR concentration above 1.45 mg/L, this is approximately one SD (0.33 mg/L) above the mean sTfR concentration (1.19 mg/L), was associated with a 3-fold increased risk of vaginosis-like microflora (RR = 3.0, 95%CI: 1.4–6.7), and the risk did not change when accounting for potential differences in the distributions of maternal age, gestational age, body mass index, parity, CRP, and smoking habits.

Albeit sTfR is considered a marker of both iron status and erythropoiesis, sTfR acts as a marker of erythropoiesis only when iron stores are adequate and sTfR additionally becomes a marker of iron status in the setting of tissue iron deficiency with or without adequate iron stores [31]. In this cohort of pregnant women, the sTfR distribution among women presenting with disturbed vaginal flora was significantly skewed to the right partly in the absence of overt iron deficiency as measured by serum ferritin. We therefore accounted for the log_10 _[sTfR/ferritin] index, which is considered the most accurate measure of body storage iron available [32,33] and documented a highly significant difference in the log_10 _[sTfR/ferritin] distributions between the normal and disturbed vaginal microflora groups of women. We therefore believe that the observed risk of vaginosis-like microflora associated with increased sTfR concentrations can reliably be attributed to insufficiently available body storage iron and cellular or tissue iron deficiency during early pregnancy.

### Documenting the association between iron and vaginal microflora status during pregnancy depends on the accuracy of the iron assays applied

Serum transferrin receptor synthesis is up-regulated in iron-deprived tissues and it can therefore be argued that assessment of iron status at the tissue level is of more functional importance when examining the effects of iron depletion on disease occurrence than conventional assessment of iron stores. Several recent studies have shown that sTfR is a very sensitive and specific index of iron deficiency during pregnancy [34,35], that sTfR assaying is superior to conventional methods for the assessment of iron status [32,33] and that the accuracy with which iron deficiency can be diagnosed is further increased by combining sTfR and ferritin [31-33]. In contrast, conventional indicators of iron status, such as red blood cell indices and markers of iron transport tend to be less sensitive or are altered by gestation independently of iron status [31,34], and therefore also have low specificity. Markers of iron storage and transport, ferritin and transferrin, may also act as acute phase reactants which may further intricate the interpretation of their values with regard to iron status, though there was no evidence of confounding by inflammation in our study.

Consequently, it may not be surprising that the array of conventional iron and red blood cell assays that were performed in our study did not pick up the association with vaginal microflora status. Ferritin is however, a very early marker in the setting of inadequate iron supplies and could therefore reasonably be expected to reflect the observed association as well, though not obvious from this study. A possible explanation is that our study lacked the power to demonstrate the effect of iron deficiency as assessed by ferritin alone, due to its biological variability, considering serum ferritin shows a wide range of values within the normal range.

If anything, it cannot be ignored that when accounting for both ferritin and soluble transferrin receptor concentrations in the log_10 _[sTfR/ferritin] index as an established highly accurate measure of body storage iron, the difference between the healthy and disturbed vaginal microflora groups of women became even more apparent than when accounting for sTfR alone.

### Iron deficiency in early pregnancy may ensue from critical preconceptional iron supplies which are further compromised by iron restriction during early pregnancy

Soluble transferrin concentrations have been shown to be steadily low during early pregnancy [31,34,36,37] and relatively decreased as compared to prepregancy concentrations by some [31] or at least not significantly elevated from the non-pregnant state until the second trimester by others [37]. This has been attributed to blunted erythropoietin production resulting in decreased erythropoiesis [36] with low peripheral reticulocyte counts during the first pregnancy trimester [37].

Decreased erythropoiesis has in turn been considered to concur with decreased iron requirements during early pregnancy due to cessation of menstrual losses [38]. Yet, the biological significance of this relative erythropoietic quiescence has not been fully explained.

It is likely that the disproportionate increases in maternal plasma volume and red cell mass leading to the physiologic anaemia of pregnancy from early gestation until term represent an important hemodynamic and hemostatic protective feature of normal pregnancy [39]. Several studies have demonstrated unfavourable pregnancy outcomes associated with high Hgb concentrations early in pregnancy, as well as in situations where Hgb concentrations fail to decline in the mid-trimester [40]. Consequently, the relative erythropoietic slowdown during early pregnancy might be targeted at rapid enhancement of such hemodilution. For instance, by the end of the first trimester, maternal plasma volume has expanded by some 15% on average [41], though not paralleled by a concomitant boost in erythropoietin release [36], as would be expected [42].

Of note is that intestinal iron absorption during the first trimester is also reduced [38], while rapidly increasing after that time with the amount of dietary iron transferred to the foetus regulated in response to maternal iron status at the level of the gut [43]. It is therefore plausible that restrictive iron absorption during early pregnancy at the time of critical processes such as placental development and organogenesis also concurs with other protective mechanisms, notably anti-oxidant and anti-infectious defence [28,40].

Against this background, it is conceivable that women entering pregnancy with impending iron deficiency may share a conflict of interest between these potentially protective iron- and erythropoiesis-restricting mechanisms on one hand, and insufficient iron availability to comply with increased basic metabolic rates and increased iron and oxygen demands on the other hand. It has been recognized that the high gestational iron needs during the second and third trimesters are met by increasing intestinal iron absorption rates beyond the first trimester up to term, but also by mobilizing available prepregnancy reserves [34]. High STfR concentrations during early pregnancy among a subset of women in this cohort may therefore reflect cellular iron deficiency as a result of subclinical or latent prepregnancy iron shortage.

### Iron deficiency and the host response against vaginal bacterial colonization

As it comes down to the biological plausibility of the observed association, it is conceivable that iron deficiency may affect both innate and cellular immune responsiveness [26,27] at the level of the vaginal mucosa, though we did not identify any previous study on micronutrient status and bacterial vaginosis. The significant association of iron deficiency anaemia with infection has however extensively been demonstrated and most importantly, these effects have been attributed to the adverse effects of iron deficiency on the immune system, even in the setting of mild micronutrient deficiencies [26,27,29]. Interestingly, our observations on the association between iron deficiency and bacterial vaginosis concur with the consistent association between maternal iron deficiency in early pregnancy and a greater risk of preterm delivery [28,29] on one hand, and with the strong and consistent association between bacterial vaginosis in early pregnancy and preterm delivery [11,12,20] on the other hand.

### Limitations of the study

Our results should be taken with caution considering our sample size was limited. Therefore our findings undeniably need to be confirmed in much larger, prospective cohort studies, preferably including non-pregnant women as well. Though the present case-control study was actually nested within a larger cohort study, failure to consistently follow-up patients enrolled at entry to prenatal care hampered any further conclusions being drawn that might be of interest to our results presented above.

As to confounding, we were able to control for most established confounders that may impinge on vaginal microflora status and on sTfR concentrations, including age, gestational length, BMI, smoking habits, and parity. Among these body mass index and parity warrant particular scrutiny, considering these variables were significantly associated with vaginal microflora status in our series, while also being known determinants of maternal sTfR concentrations. Maternal sTfR concentrations were however not significantly correlated with BMI (p = 0.4). The correlation between sTfR and parity was also not apparent from our data, this is, when parity was handled as the raw, categorical variable (p = 0.2), yet the correlation became highly significant when parity was handled as a binary variable (0.005). Therefore, it cannot be ignored that the observed association between maternal sTfR concentrations and vaginal microflora status concurs with the association between sTfR and parity. In particular, we found that multiparous women had on average a significantly higher sTfR (p = 0.003) and were also significantly more likely to have disturbed vaginal microflora (p = 0.04) as compared to nulliparous women. Though these interactions were cancelled in the multivariable analysis by the correlation between sTfR and vaginal microflora status, it still needs to be considered that parity may act as a confounder to the former association. If anything, as it is only plausible that parity affects mean sTfR rather than sTfR concentrations determining parity, the most conceivable explanation would be that sTfR concentrations are the explanatory variable to the association between parity and vaginal microflora status. Ethnicity was not a confounder to our study as all women were of white Caucasian origin. We did not collect any data on sexual behaviour-related characteristics nor on vaginal douching, which have consistently been associated with BV, and therefore it cannot be precluded that differential sexual behaviour and differences in use of vaginal hygiene products between both groups may have confounded our results at least to some extent.

It should also be acknowledged that, owing to the design of the study, we are also unaware of the timing of the exposure relative to the timing of the outcome. There is good evidence from the literature however, that the sTfR concentrations at these early gestational ages most likely reflect preconceptional sTfR concentrations, as discussed above.

Finally, at the sTfR cut-off level chosen, the strength of the association was convincingly strong and this was further reflected by the high specificity and the high NPV indicating that above this threshold, women with disturbed vaginal microflora were overrepresented among subjects with a high sTfR and hence with impaired iron availability or tissue iron needs. On the contrary, though subjects with sTfR concentrations below the threshold represented the preponderance of women with normal vaginal microflora, a substantial proportion of the women with disturbed vaginal microflora also had no evidence of iron deficiency, and therefore the sensitivity and the PPV were actually low. This observation is not unexpected to the extent BV – much alike most conditions, is thought of as a multifactorial condition that obviously can occur in the absence of iron deficiency.

### Iron supplementing and bacterial vaginosis: an opportunity for preconceptional prevention of adverse pregnancy outcome?

We could not establish a significant relationship between iron supplementing and various indicators of iron deficiency in this population with the exception of a marginally significant association with ferritin values, but this finding should be taken with caution considering we had no information on duration of iron supplementing preceding iron status assessment. However, since study subjects were enrolled at their first antenatal visit, the time span between commencement of iron supplementing and iron status assessment must have been rather short. If anything, our study lacked the power to substantiate such an association considering the prevalence of iron deficiency was fairly low and rates of supplemental iron intake rather high. Previous studies did demonstrate a significant decrease of sTfR concentrations following iron administration among both pregnant [34] and non-pregnant subjects [44].

From the above notions on iron metabolism in early pregnancy it may be inferred however, that adequate preconceptional iron stores and therefore preconceptional iron supplementing rather than first trimester supplements may better serve the goal of preventing BV-associated adverse pregnancy outcome and preterm birth in particular. Of note is that in animal models, preconceptional nutrional status has recently also been associated with non-infectious preterm birth [45].

## Conclusion

We conclude that subclinical iron deficiency, presumably resulting from inadequate preconceptional iron supplies, is strongly and independently associated with vaginosis-like microflora during early pregnancy, after accounting for maternal age, gestational length, body mass index, parity, CRP, and smoking habits as potential confounders. The strong association between tissue iron deficiency with vaginosis-like flora during early pregnancy was documented by assessment of highly sensitive and specific markers, in particular maternal soluble transferrin receptors and the log_10 _[sTfR/ferritin] iron deficiency index, while the association was not apparent from conventional iron and red blood cell indicators.

These findings need to be confirmed in larger, prospective cohort studies, preferably including non-pregnant women as well. If so, the association of latent, subclinical or functional iron deficiency with bacterial vaginosis may be of paramount interest to the primary and secondary prevention of bacterial vaginosis and bacterial vaginosis-associated disease, including preterm birth.

## Competing interests

The author(s) declare that they have no competing interests.

## Authors' contributions

HV and MT participated in the development of the study design, the collection of the study samples, the collection, analysis and interpretation of the data, and in the writing of the report. JD participated in the development of the study design, the analysis of the blood samples, the interpretation of the data, and provided important intellectual content to the manuscript. KR and SB participated in the collection of the study samples, the collection of the data, and provided important intellectual content to the manuscript. GC participated in the development of the study design, conducted the analysis of all vaginal samples, and provided important intellectual content to the manuscript.

## Pre-publication history

The pre-publication history for this paper can be accessed here:


